# Supplementary material for: Structure of the lens MP20 mediated adhesive junction
Source: Nat Commun. 2025 Mar 26;16:2977. doi: 10.1038/s41467-025-57903-6 (PMC11947226; doi:10.1038/s41467-025-57903-6)
Supplement: Supplementary file 1 — Supplementary Information [file 41467_2025_57903_MOESM1_ESM.pdf]

SUPPLEMENTARY INFORMATION

Structure of the lens MP20 mediated adhesive junction

William J. Nicolas[1,2], Anna Shiriaeva[1*], Michael W. Martynowycz[1*], Angus C Grey[3], Yasmeen Ruma[1,2], Paul J Donaldson[3] and Tamir Gonen[1,2,4$]*

[1]Department of Biological Chemistry, David Geffen School of Medicine, University of California, Los Angeles, CA 90095

[2]Howard Hughes Medical Institute, University of California, Los Angeles, CA 90095

[3]Department of Physiology, School of Medical Sciences, University of Auckland, Auckland, NZ

[4]Department of Physiology, David Geffen School of Medicine, University of California, Los Angeles, CA 90095

[*] These authors contributed equally to the work

[$]Correspondence: tgonen@g.ucla.edu

Supplementary Figure 1: Purification of MP20 protein for crystallization

a. MP20 primary amino acid sequence used for expression and MP20 purification for crystallization. b. High Performance Liquid Chromatography (HPLC) profile (dead volume was cut out). The MP20 absorbance peak can be seen at 27mL elution volume (red dashed line). c. SDS-PAGE of the peak with visible bands at MP20 molecular weight.

Supplementary Figure 1
Purification of MP20

a

MP20 a.a. sequence used

MKTIIALSYIFCLVFADYKDDDDAKLQTMHHHHHHHHHHLEVLFQMYSFMGGGLFCAWVGTILLVVAMATDHWMQYRLSGSFAHQGLWRYCLGNKCYLQTDSIAYWNATR
AFMILSALCAISGIIMGIMAFAHQPTFSRISRPFSAGIMFFSSTLFVVLALAIYTGVTVSFLGRRFGDWRFSWSYILGWVAVLMTFFAGIFYMCAYRVHECRRLSTPR*

b

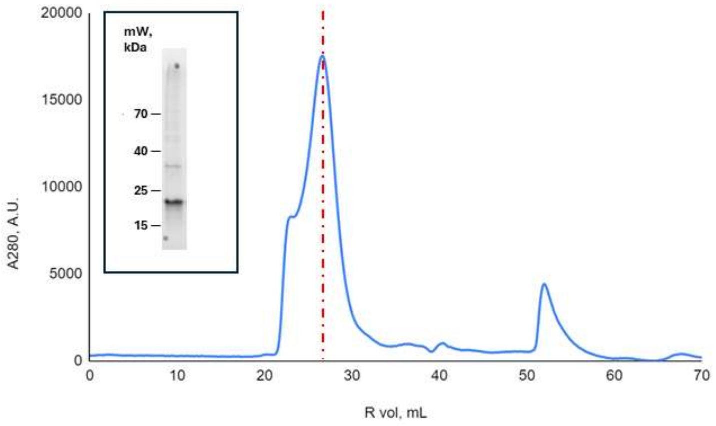

Supplementary Figure 2: Packing of MP20 crystals

a. Side view of MP20 crystal octamer mediated by the loop interactions. b. Top view of the MP20 crystal octamer

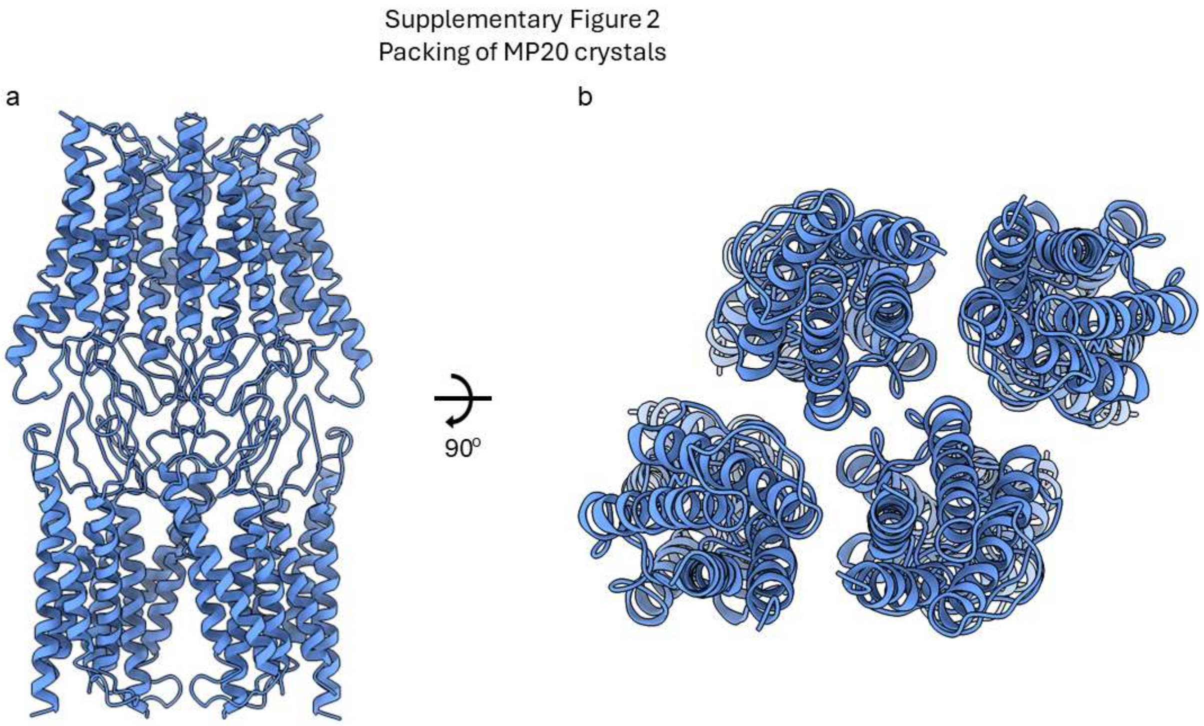

Supplementary Figure 3: Adhesive interactions in the head-head MP20 junctions

a. Side view of tetramers inserted in their respective membrane bilayers interacting with an opposing tetramer, forming an octamer. One interaction involving two apposed protomers is highlighted (yellow and blue). Lateral chains involved in the putative interactions are made visible in the loops. Loops and N/C-termini are labelled. b. Magnified view of the red inset in a. with a 90° rotation of the molecule towards the right, showing the interface between the ECLs from the protomers involved in the “hand shake” interaction. ECLs are also labelled. Putative intra, inter-chain electrostatic interactions and di-sulfide bridges are shown in purple, green and yellow dashed lines, respectively. The amino acids involved in those putative interactions are labelled. c. 90° rotation of the molecule in b. downward showing the side-by-side interactions between two protomers of a dimer within one tetramer. The amino acids involved in those putative interactions are labelled and traced in green. The protomers in white belong to the bottom and top tetramer shown in a. and interact with the yellow and blue protomer, respectively.

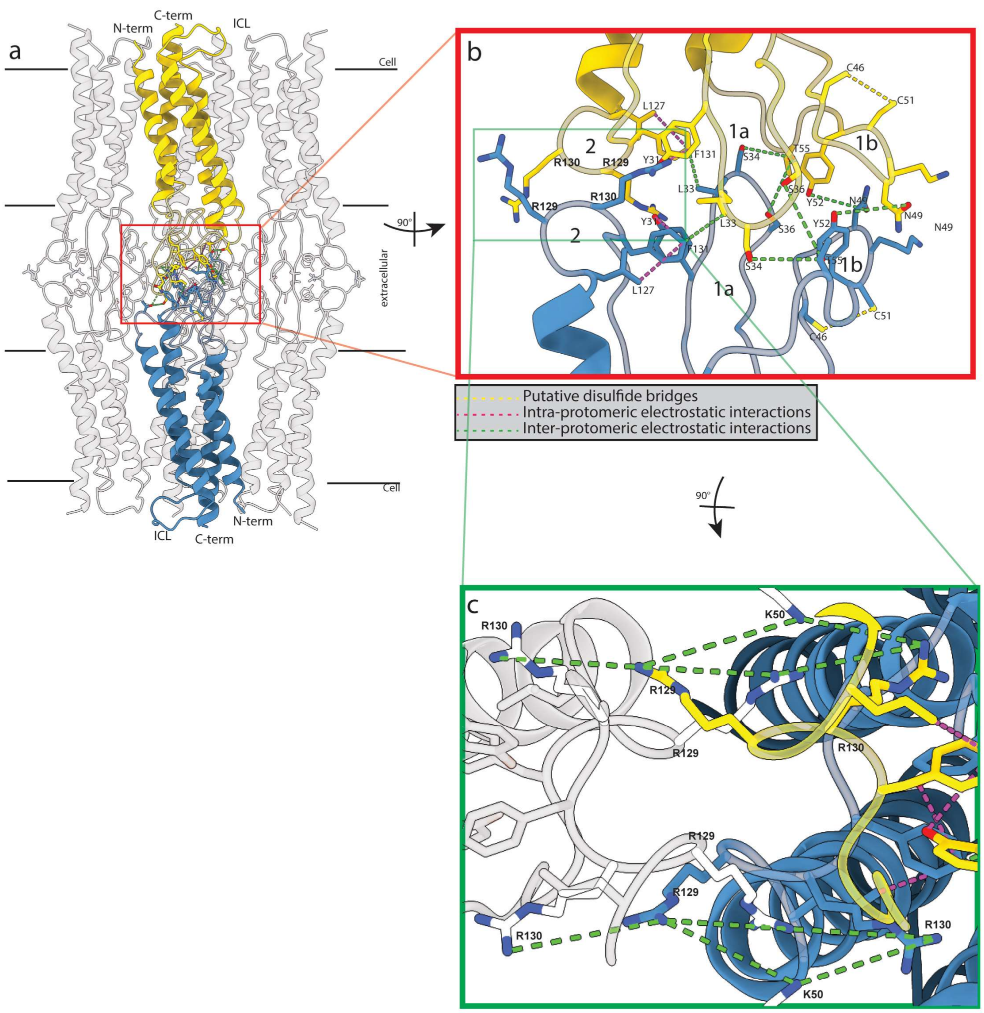

Supplementary Figure 4

Alphafold3 predictive models for lens MP20. a. Predictive model of the monomer. b.predicted model of a dimer. c. Predicted model of an octamer.

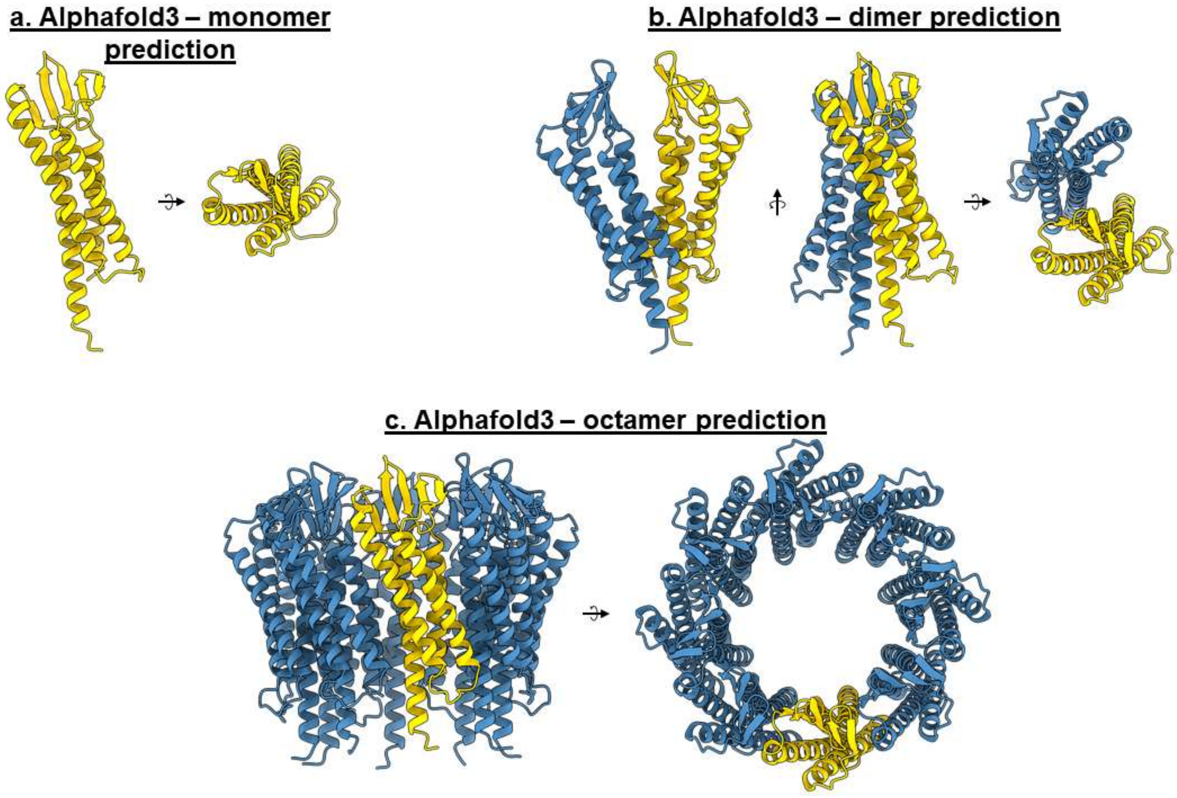

SUPPLEMENTARY MOVIES LEGENDS

Supplementary Movie 1

MicroED dataset diffracting to 3.25Å acquired on an MP20 crystalline lamella.

Supplementary Movie 2

360° rotational video of the MP20 monomer model in rainbow colors.

Supplementary Movie 3

360° rotational video of two MP20 monomers involved in a head-head interaction, in rainbow colors.

Supplementary Movie 4

360° rotational video of two MP20 tetramers (yellow and blue) involved in head-head interaction, forming an octamer.

Supplementary Movie 5

360° rotational video of an MP20 octamer showing its steric hindrance.

Supplementary Movie 6

Detailed rocking view of the head-head interaction with the lateral chains involved in putative contacts mentioned in manuscript made visible. Putative intra, inter-chain electrostatic interactions and di-sulfide bridges are shown by protomer color (yellow or blue), green and red dashed lines.
