# Supplementary material for: Structure of the lens MP20 mediated adhesive junction
Source: Nat Commun. 2025 Mar 26;16:2977. doi: 10.1038/s41467-025-57903-6 (PMC11947226; doi:10.1038/s41467-025-57903-6)
Supplement: Supplementary file 2 — Description of Additional Supplementary Files [file 41467_2025_57903_MOESM2_ESM.pdf]

Description of Additional Supplementary Files

File name: Supplementary Movie 1
Description: MicroED dataset diffracting to 3.25Å acquired on an MP20 crystalline lamella.

File name: Supplementary Movie 2
Description: 360° rotational video of the MP20 monomer model in rainbow colors.

File name: Supplementary Movie 3
Description: 360° rotational video of two MP20 monomers involved in a head-head interaction, in rainbow colors.

File name: Supplementary Movie 4
Description: 360° rotational video of two MP20 tetramers (yellow and blue) involved in head-head interaction, forming an octamer.

File name: Supplementary Movie 5
Description: 360° rotational video of an MP20 octamer showing its steric hindrance.

File name: Supplementary Movie 6
Description: Detailed rocking view of the head-head interaction with the lateral chains involved in putative contacts mentioned in manuscript made visible. Putative intra, inter-chain electrostatic interactions and di-sulfide bridges are shown by protomer color (yellow or blue), green and red dashed lines.
