# Supplementary material for: Structure of the lens MP20 mediated adhesive junction
Source: Nat Commun. 2025 Mar 26;16:2977. doi: 10.1038/s41467-025-57903-6 (PMC11947226; doi:10.1038/s41467-025-57903-6)
Supplement: Supplementary file 9 — Reporting Summary [file 41467_2025_57903_MOESM9_ESM.pdf]

nature portfolio

Corresponding author(s): Tamir Gonen

Last updated by author(s): Jun 3, 2024

Reporting Summary

Nature Portfolio wishes to improve the reproducibility of the work that we publish. This form provides structure for consistency and transparency in reporting. For further information on Nature Portfolio policies, see our Editorial Policies and the Editorial Policy Checklist.

Statistics

For all statistical analyses, confirm that the following items are present in the figure legend, table legend, main text, or Methods section.

n/a	Confirmed	
☐	☒	The exact sample size (*n*) for each experimental group/condition, given as a discrete number and unit of measurement
☒	☐	A statement on whether measurements were taken from distinct samples or whether the same sample was measured repeatedly
☒	☐	The statistical test(s) used AND whether they are one- or two-sided<br>*Only common tests should be described solely by name; describe more complex techniques in the Methods section.*
☒	☐	A description of all covariates tested
☒	☐	A description of any assumptions or corrections, such as tests of normality and adjustment for multiple comparisons
☒	☐	A full description of the statistical parameters including central tendency (e.g. means) or other basic estimates (e.g. regression coefficient) AND variation (e.g. standard deviation) or associated estimates of uncertainty (e.g. confidence intervals)
☒	☐	For null hypothesis testing, the test statistic (e.g. *F*, *t*, *r*) with confidence intervals, effect sizes, degrees of freedom and *P* value noted<br>*Give P values as exact values whenever suitable.*
☒	☐	For Bayesian analysis, information on the choice of priors and Markov chain Monte Carlo settings
☒	☐	For hierarchical and complex designs, identification of the appropriate level for tests and full reporting of outcomes
☒	☐	Estimates of effect sizes (e.g. Cohen's *d*, Pearson's *r*), indicating how they were calculated

Our web collection on statistics for biologists contains articles on many of the points above.

Software and code

Policy information about availability of computer code

Data collection	SerialEM, ThermoFisher TUI
Data analysis	mrc2smv (https://cryoem.ucla.edu/downloads/snapshots), XDS, Phenix, (Win)COOT, ChimeraX

For manuscripts utilizing custom algorithms or software that are central to the research but not yet described in published literature, software must be made available to editors and reviewers. We strongly encourage code deposition in a community repository (e.g. GitHub). See the Nature Portfolio guidelines for submitting code & software for further information.

Data

Policy information about availability of data

All manuscripts must include a data availability statement. This statement should provide the following information, where applicable:

- Accession codes, unique identifiers, or web links for publicly available datasets
- A description of any restrictions on data availability
- For clinical datasets or third party data, please ensure that the statement adheres to our policy

Raw .eer microED datasets used for the MP20 structure are available upon request. PDB structures will be available after reviewing.

Research involving human participants, their data, or biological material

Policy information about studies with human participants or human data. See also policy information about sex, gender (identity/presentation), and sexual orientation and race, ethnicity and racism.

Reporting on sex and gender	N/A
Reporting on race, ethnicity, or other socially relevant groupings	N/A
Population characteristics	N/A
Recruitment	N/A
Ethics oversight	All human lens work was conducted in compliance with the Declaration of 402 Helsinki and was approved by the Northern X Regional ethics Committee (Ref: NTX/07/08/079).

Note that full information on the approval of the study protocol must also be provided in the manuscript.

Field-specific reporting

Please select the one below that is the best fit for your research. If you are not sure, read the appropriate sections before making your selection.

☒ Life sciences ☐ Behavioural & social sciences ☐ Ecological, evolutionary & environmental sciences

For a reference copy of the document with all sections, see nature.com/documents/nr-reporting-summary-flat.pdf

Life sciences study design

All studies must disclose on these points even when the disclosure is negative.

Sample size	3 human lens was used for the fluorescent study of the insertion of MP20 in the membrane of lens cells (Figure 4)
Data exclusions	24 datasets were acquired from MP20 milled crystals, the 6 best, yielding the best statistics, were used for the presented structure
Replication	Biological replicates = 3 human lenses (63y M, 46y M, 71 y M) were used for the labeling.
Randomization	Determination of milling targets was done based on the position of the crystal on the grid, not the shape nor size of the crystal.
Blinding	N/A

Reporting for specific materials, systems and methods

We require information from authors about some types of materials, experimental systems and methods used in many studies. Here, indicate whether each material, system or method listed is relevant to your study. If you are not sure if a list item applies to your research, read the appropriate section before selecting a response.

Materials & experimental systems

n/a	Involved in the study	
☐	☒	Antibodies
☐	☒	Eukaryotic cell lines
☒	☐	Palaeontology and archaeology
☐	☒	Animals and other organisms
☒	☐	Clinical data
☒	☐	Dual use research of concern
☒	☐	Plants

Methods

n/a	Involved in the study	
☒	☐	ChIP-seq
☒	☐	Flow cytometry
☒	☐	MRI-based neuroimaging

Antibodies

Antibodies used	HRP-conjugated anti-his antibody (GenScript) for MP20 Western blot (Fig. 1).<br>Carboxyl tail specific MP20 antibody + secondary fluorescein-419 conjugated antibody (Santa Cruz Biotechnology, Santa Cruz, California, USA).

Validation	HRP anti-his antibody and fluorescein-419 antibody are commercially available and validated by the company.<br>Carboxy tail specific MP20 antibody was created and validated as described in the following study: MARK L. ARNESON, CHARLES F. LOUIS,<br>Structural Arrangement of Lens Fiber Cell Plasma Membrane Protein MP20,<br>Experimental Eye Research,<br>Volume 66, Issue 4,<br>1998 (https://www.sciencedirect.com/science/article/pii/S0014483598904778?via=ihub)

Eukaryotic cell lines

Policy information about cell lines and Sex and Gender in Research

Cell line source(s)	SF9 insect cells from organism Spodoptera Frugiperda, purchased at Expression Systems
Authentication	Cell lines used were commercially purchased
Mycoplasma contamination	Cell lines tested negative for mycoplasma contamination
Commonly misidentified lines<br>(See ICLAC register)	N/A

Animals and other research organisms

Policy information about studies involving animals; ARRIVE guidelines recommended for reporting animal research, and Sex and Gender in Research

Laboratory animals	N/A
Wild animals	N/A
Reporting on sex	N/A
Field-collected samples	N/A
Ethics oversight	Northern X Regional ethics Committee (Ref: NTX/07/08/079).

Note that full information on the approval of the study protocol must also be provided in the manuscript.

Plants

Seed stocks	N/A
Novel plant genotypes	N/A
Authentication	N/A
